# Automated Analysis of Stereotypical Movements in Videos of Children With Autism Spectrum Disorder

**DOI:** 10.1001/jamanetworkopen.2024.32851

**Published:** 2024-09-12

**Authors:** Tal Barami, Liora Manelis-Baram, Hadas Kaiser, Michal Ilan, Aviv Slobodkin, Ofri Hadashi, Dor Hadad, Danel Waissengreen, Tanya Nitzan, Idan Menashe, Analya Michaelovsky, Michal Begin, Ditza A. Zachor, Yair Sadaka, Judah Koler, Dikla Zagdon, Gal Meiri, Omri Azencot, Andrei Sharf, Ilan Dinstein

**Affiliations:** 1 Department of Computer Science, Ben-Gurion University of the Negev, Beer Sheva, Israel; 2 Azrieli National Centre for Autism and Neurodevelopment Research, Ben Gurion University of the Negev, Beer Sheva, Israel; 3 Department of Psychology, Ben-Gurion University of the Negev, Beer Sheva, Israel; 4 Department of Cognitive and Brain Sciences, Ben-Gurion University of the Negev, Beer Sheva, Israel; 5 Department of Public Health, Ben-Gurion University of the Negev, Beer Sheva, Israel; 6 Department of Computer Science, Bar-Ilan University, Ramat Gan, Israel; 7 Pre-School Psychiatry Unit, Soroka University Medical Center, Beer Sheva, Israel; 8 Zusman Child Development Center, Soroka University Medical Center, Beer Sheva, Israel; 9 Child Development Center, Leumit Healthcare Services, Jerusalem, Israel; 10 The Autism Center/ ALUT, Shamir (Assaf Harofeh) Medical Center, Be’er Ya’akov, Israel; 11 Faculty of Medicine, Tel Aviv University, Tel Aviv, Israel; 12 Neuro-Developmental Research Centre, Beer Sheva Mental Health Centre, Ministry of Health, Beer Sheva, Israel; 13 Seymour Fox School of Education, The Hebrew University of Jerusalem, Jerusalem, Israel

## Abstract

**Importance:**

Stereotypical motor movements (SMMs) are a form of restricted and repetitive behavior, which is a core symptom of autism spectrum disorder (ASD). Current quantification of SMM severity is extremely limited, with studies relying on coarse and subjective caregiver reports or laborious manual annotation of short video recordings.

**Objective:**

To assess the utility of a new open-source AI algorithm that can analyze extensive video recordings of children and automatically identify segments with heterogeneous SMMs, thereby enabling their direct and objective quantification.

**Design, Setting, and Participants:**

This retrospective cohort study included 241 children (aged 1.4 to 8.0 years) with ASD. Video recordings of 319 behavioral assessments carried out at the Azrieli National Centre for Autism and Neurodevelopment Research in Israel between 2017 and 2021 were extracted. Behavioral assessments included cognitive, language, and autism diagnostic observation schedule, 2nd edition (ADOS-2) assessments. Data were analyzed from October 2020 to May 2024.

**Exposures:**

Each assessment was recorded with 2 to 4 cameras, yielding 580 hours of video footage. Within these extensive video recordings, manual annotators identified 7352 video segments containing heterogeneous SMMs performed by different children (21.14 hours of video).

**Main outcomes and measures:**

A pose estimation algorithm was used to extract skeletal representations of all individuals in each video frame and was trained an object detection algorithm to identify the child in each video. The skeletal representation of the child was then used to train an SMM recognition algorithm using a 3 dimensional convolutional neural network. Data from 220 children were used for training and data from the remaining 21 children were used for testing.

**Results:**

Among 319 behavioral assessment recordings from 241 children (172 [78%] male; mean [SD] age, 3.97 [1.30] years), the algorithm accurately detected 92.53% (95% CI, 81.09%-95.10%) of manually annotated SMMs in our test data with 66.82% (95% CI, 55.28%-72.05%) precision. Overall number and duration of algorithm-identified SMMs per child were highly correlated with manually annotated number and duration of SMMs (*r* = 0.8; 95% CI, 0.67-0.93; *P* < .001; and *r* = 0.88; 95% CI, 0.74-0.96; *P* < .001, respectively).

**Conclusions and relevance:**

This study suggests the ability of an algorithm to identify a highly diverse range of SMMs and quantify them with high accuracy, enabling objective and direct estimation of SMM severity in individual children with ASD.

## Key Points

**Question:**

Is it possible to train a deep learning algorithm to accurately identify and quantify stereotypical motor movements (SMMs) in video recordings of children with autism?

**Findings:**

In this cohort study of 319 behavioral assessment recordings from 241 children, an algorithm was trained and tested with the largest video dataset of children with autism spectrum disorder (ASD) curated to date. The algorithm successfully detected 92.53% of manually identified SMMs with 66.82% precision, achieving highly accurate quantification of SMMs per child that were strongly correlated with quantification by manual annotation.

**Meaning:**

These findings suggest the utility of the tested algorithm for objective and direct quantification of SMM severity in children with ASD, offering a new freely available, open-source algorithm and dataset that enable transformative basic and clinical ASD research.

## Teaser

This cohort study evaluates the utility of a new open-source artificial intelligence algorithm that can analyze extensive video recordings of children with autism spectrum disorder (ASD) and automatically identify segments with heterogeneous stereotypical motor movements.

## Introduction

Stereotypical motor movements (SMMs) are apparent in approximately 50% of individuals with autism spectrum disorders (ASD).^1^ They embody a form of restricted and repetitive behaviors, which are a core symptom of ASD.^2^ SMMs have been defined as repetitive, rhythmical, coordinated, seemingly purposeless movements^1,3,4,5^ that can be categorized into groups according to body topography,^6^ complexity,^7,8^ and/or function.^9^ Common examples of SMMs include hand flapping, body rocking, jumping, and turning in circles. Although SMMs are not unique to ASD, they are more prevalent in ASD than in other developmental disorders.^3^

SMMs are often described by individuals with ASD as an adaptive self-regulating coping mechanism for situations involving sensory overload, anxiety, or excitement.^10,11,12,13^ However, frequent SMMs may also disrupt learning, skill acquisition, and social communication.^14,15,16,17^ Regardless of their function, identifying and quantifying SMMs is important for assessing SMM severity at diagnosis, estimating changes their severity over time, performing comparisons across developmental disorders, and for studying their underlying neurophysiology.

Most studies to date have measured SMMs using parent questionnaires such as the Repetitive Behaviors Scale-Revised.^18^ Although parent questionnaires provide an important perspective on SMMs, their accuracy and sensitivity are limited due to narrow scoring ranges and possible reporter bias.^19^ One alternative is to measure SMMs in video recordings by manually annotating them.^6,8,20,21^ While this approach allows direct quantification of SMMs, it is laborious, requires expertise, and is therefore not scalable.^6,20^ Another alternative is to identify SMMs by analyzing accelerometry data recorded with small sensors placed on multiple body locations. Several studies have demonstrated high accuracy in identifying predefined SMMs^22,23,24^ that were manually annotated in simultaneous video recordings. While this is an exciting approach, further research is necessary to extend findings beyond the 6 participants recorded and analyzed in these studies.

Additional studies have applied machine-learning techniques to classify SMM types using Kinect^25,26^ (depth camera) recordings of SMMs performed in a laboratory setting or short video recordings (approximately 90 seconds) of SMMs captured by parents at home.^27,28,29^ These studies, however, only attempted to distinguish between 3 to 4 predefined SMMs captured in short recordings, rather than identifying heterogeneous SMMs that occur infrequently within long recordings of naturally behaving children with ASD. Note that clinicians typically monitor the existence and frequency of SMMs rather than their specific identity.

Developing automated computer vision tools for identifying and quantifying SMMs in extensive video recordings would be transformative for the field, enabling direct, objective, scalable, high-throughput, low-effort quantification of this core ASD symptom. Achieving this task, however, requires overcoming 2 key challenges. First, most recordings contain more than 1 individual, making it necessary to identify and track the individual with ASD so that only their movements are analyzed. Second, SMMs are highly heterogeneous, with individuals with ASD displaying distinct and unique SMMs.^30,31^ To accurately identify and quantify SMMs, it is, therefore, critical to train algorithms on large, well-annotated datasets that include both heterogeneous SMM exemplars and heterogeneous movements that are not SMMs.

To overcome these challenges, we curated the largest SMM dataset we know of to date, composed of video recordings from 319 clinical assessments of 241 children. We extracted the skeletal representation of all individuals visible in each frame, yielding a compact, sparse, and efficient representation of their body movements. We then manually identified 7352 video segments where the child exhibited an SMM. This library of SMMs was used to train and test the algorithm. The objective of this study was to demonstrate the utility of the algorithm for analyzing extensive video recordings of children and automatically identifying segments with heterogeneous SMMs. We intentionally targeted all SMMs rather than specific types or classes of SMMs given that different researchers may want to subgroup SMMs using different criteria.

## Methods

This manuscript follows the Strengthening the Reporting of Observational Studies in Epidemiology (STROBE) reporting guideline. The Helsinki committees at Soroka University Medical Center (SUMC), Shamir Medical Center, and Leumit Healthcare Services as well as the internal review board of the Hebrew University approved this study. Parents of all participating children signed an informed consent form.

### Participants

We analyzed video recordings from 241 children, aged 1.4 to 8.0 years, who were recruited between 2017 and 2021 at the Azrieli National Centre for Autism and Neurodevelopment Research (ANCAN), a collaboration between Ben-Gurion University of the Negev and 8 clinical sites throughout Israel. The ANCAN autism database includes, among other measures, video recordings of clinical assessments.^32,33^ We selected children with a diagnosis of ASD according to *Diagnostic and Statistical Manual of Mental Disorders* (Fifth Edition) criteria^2^ who completed an Autism Diagnostic Observation Schedule, 2nd edition (ADOS-2) assessment^34^ where they scored 2 or more on ADOS-2 item D2 (repetitive hand/finger movements) or item D4 or D5 (stereotypical behavior in ADOS-2 modules 1 and 2/Toddlers, respectively).

### Behavioral Assessment Recordings and Computing Hardware

We analyzed 883 video recordings from 319 behavioral assessments of 241 children. Each child contributed 1 to 2 assessments. These included 226 ADOS-2^34^; 71 Preschool Language Scale, 4th edition (PLS-4)^35^; and 22 developmental or cognitive assessments composed of 11 Mullen Scales of Early Learning^36^ and 11 Wechsler Preschool and Primary Intelligence, Third Edition^37^ assessments. Behavioral assessment rooms were equipped with 2 to 4 video cameras with a resolution of 1080 by 1920 pixels, recording at 30 frames per second. Algorithm development, including all training and testing, was performed with 2 RTX3090 GPUs (NVIDIA), a 32-core 3.5GHz Ryzen Threadripper PRO 3975WX CPU (AMD), and 264GB of RAM.

### Pose Estimation and Skeleton Tracking

We used OpenPose^38^ to extract the locations of 17 skeletal joints in 2-dimensional space for each person in each frame. To enable easy manual annotation of the child’s skeleton (see next section), it was necessary to label the identity of each skeleton consistently across frames. We applied an off-the-shelf spatial-temporal affinity field tracking algorithm^39^ to achieve this without performing any additional training with our data.

### Manual Annotation of SMMs and the Child’s Skeleton

SMMs were manually identified and annotated using in-house software (eFigure 1 in Supplement 1). Annotators were undergraduate students trained by a clinician with more than 15 years of experience. They viewed the videos, labeled the start and end time of each SMM, classified its type (see eTable 1 in Supplement 1), and marked the skeleton ID of the child. This resulted in a list of 7352 video segments containing SMMs that were a mean (SD) of 9.89 (9.56) seconds long. Note that all 580 hours of video recordings were examined and SMMs were found in segments corresponding to 21.14 hours (approximately 3.5% of total length). The remainder of the recordings contained movements that were not SMMs.

### Child Detection

We trained the YOLOv5^40^ object detection algorithm using a variant pretrained on the COCO object dataset^41^ to identify the child in each video frame. To train the algorithm, we first created bounding boxes around each of the skeletons per frame by maximizing their intersection over union. We sampled 30 000 frames uniformly from the 7352 manually annotated SMM video segments described previously where the child’s skeleton was manually labeled. We then cropped images from the bounding boxes of each child and adult (ie, all other) skeleton and used them as input into the algorithm. We used 80% of the data to train the algorithm and 20% to test it. The algorithm achieved 95% precision and 92% recall for correctly identifying bounding boxes containing a child. We applied the algorithm to all frames of all videos and performed all further analyses only with child skeletons (eFigure 2 in Supplement 1).

### SMM Identification Algorithm

In addition to the 7352 manually identified SMM video segments described previously, we extracted 28 648 randomly selected video segments with an equivalent distribution of lengths that contained non-SMM movements. We defined the data from the first 220 children (295 assessments) as our training dataset (6597 segments with SMMs and 24 923 without). The remaining 755 SMM segments from 21 children (24 assessments) were held out for testing. There were no significant differences in the gender, age, or behavioral scores of children in the train and test datasets (Table). We selected this relatively small test dataset because we also performed manual annotation on it twice to establish SMM interrater reliability. This ensured that our test data were thoroughly validated.

**Table.** Descriptive Statistics of Sex, Age, Autism Diagnostic Observation Schedule, 2nd Edition (ADOS-2) Scores, Cognitive Scores, and Preschool Language Scale, 4th Edition (PLS-4) Scores in the Training and Test Sets

Characteristic	Participants, No. (%)		Statistical comparison	*P* value
	Train set (n = 220)	Test set (n = 21)		
Sex				
Male	172 (78.18)	15 (71.43)	χ^2^_1_ (*N* = 241) = 0.19	.65
Female	48 (22.82)	6 (28.57)		
Primary language				
Hebrew	192 (87.28)	15 (71.43)	χ^2^_1_ (*N* = 241) = 2.77	.09
Arabic	28 (12.72)	6 (28.57)		
Age, mean (SD), y	3.97 (1.30)	4.32 (1.39)	*t*_239_ = −1.19	.24
ADOS-2, mean (SD)				
Total CSS	7.07 (2.23)	6.67 (1.96)	*t*_239_ = 0.80	.43
Social Affect CSS	6.52 (2.38)	6.33 (2.17)	*t*_239_ = 0.35	.73
Restricted Repetitive Behaviors CSS	8.01 (1.54)	7.48 (1.54)	*t*_239_ = 1.51	.13
Cognitive score				
Total No. of participants	183	16	NA	.50
Mean (SD)	76.49 (18.28)	73.31 (13.83)	*t*_214_ = 0.68	
PLS- 4				
Total No. of participants	148	9	NA	.69
Mean (SD)	68.18 (21.01)	71 (14.33)	*t*_174_ = 0.4	

Abbreviations: CSS, calibrated severity score; NA, not applicable.

We used a PoseConv3D model^42^ that was pretrained on the Kinetics-400 dataset^43^ to create the ASDMotion algorithm. Details about this algorithm and comparison with 3 alternative models are available in eTable 3 in Supplement 1. The algorithm was trained to yield a score between 0 and 1 corresponding to its confidence that a segment contained an SMM.

### Testing the Algorithm

To test the algorithm, we split each of the test video recordings into a sequence of overlapping segments with a width of 200 frames and a step size of 30 frames (ie, sliding window with 170 frame overlap). Each segment was scored by the algorithm and a frame-wise SMM score was calculated by selecting the maximum value per frame across overlapping segments (eFigure 3 in Supplement 1). All frames with a score equal to or greater than 0.85 (arbitrary threshold) were classified as containing an SMM, and contiguous SMM frames were concatenated into a single SMM movement. This yielded a list of automatically identified SMMs with their respective start and end times, enabling us to calculate the total number of SMMs, the number of SMMs per minute, and their median length per child (eTable 2 in Supplement 1).

To assess accuracy, we first calculated precision (ie, positive predictive value) and recall (sensitivity) per frame. Since SMMs are short, scarce, and variable in their timing within each video, recall and precision values expected by chance are infinitesimal. In a final analysis of 1456 video segments that were reannotated by 2 independent annotators, we also calculated negative predictive value and specificity.

### Statistical Analysis

All statistical analyses were performed using custom-written code in Python. Sex differences in precision and recall scores were evaluated using a Mann-Whitney test. Age, ADOS-2, cognitive, and PLS-4 score differences between the training and testing sets were assessed with independent sample *t* tests. Pearson, Spearman, and concordance correlation coefficients were computed to assess the correspondence between the algorithm and the manual annotation. CIs for precision and recall were calculated using the standard error of the mean. CIs for Pearson correlation coefficients were estimated by bootstrapping with 1000 resamples. Finally, interrater reliability was estimated using percentage agreement and Cohen κ. A statistical threshold of *P* < .05 was used throughout. Data were analyzed from October 2020 to May 2024.

## Results

**Figure 1.**
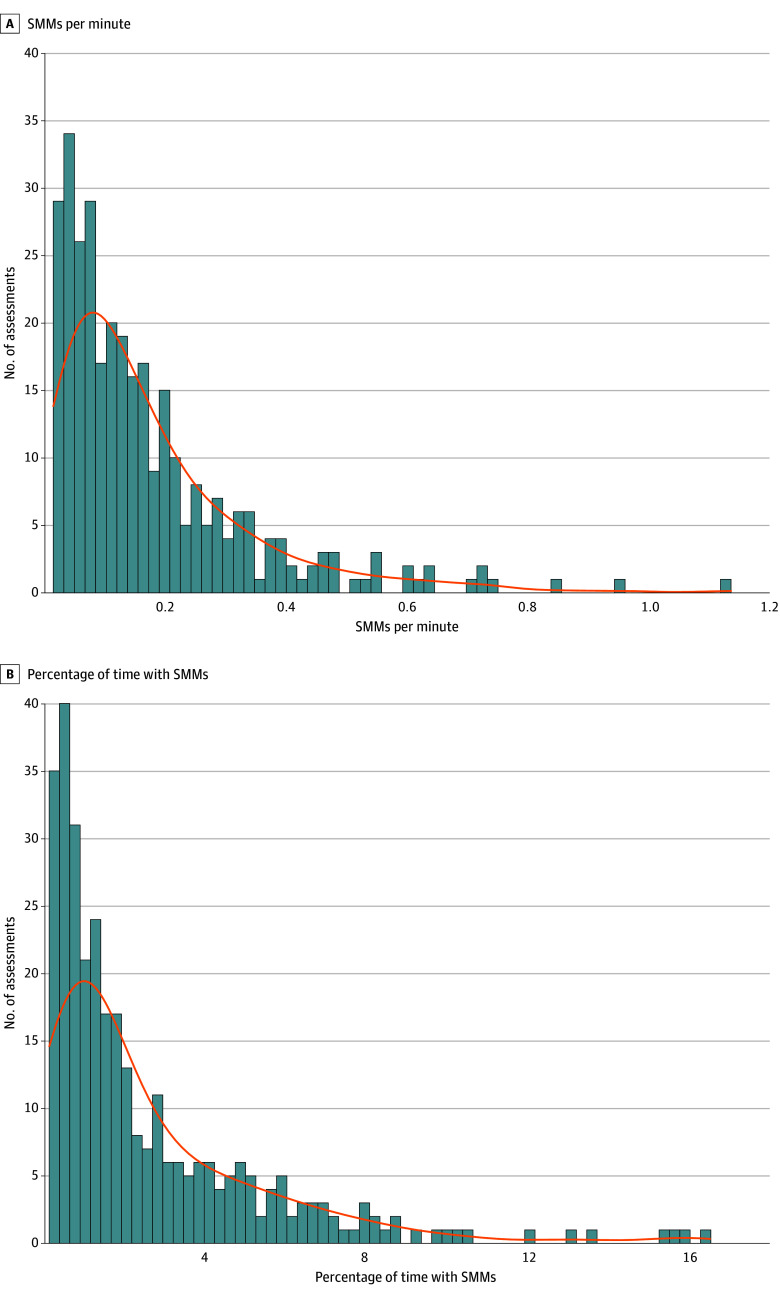
Distribution of Stereotypical Motor Movements (SMMs) Across Children and Recordings Different children with autism spectrum disorder exhibited different rates and durations of SMMs, as identified by manual annotation. Histograms demonstrate the distribution of SMMs per assessment. Orange curve indicates probability density function corresponding to each histogram.

We analyzed video recordings from 319 behavioral assessments of 241 children with ASD aged 1.4 to 8.0 years (172 [78%] male; mean [SD] age, 3.97 [1.30] years). The number and duration of SMMs, as identified by manual annotation, varied greatly across children and behavioral assessments (Figure 1; eTable 2 in Supplement 1). To adjust for different assessment lengths, we quantified the number of SMMs per minute of recording (median [IQR], 0.12 [0.06-0.22]) and the percentage of time with SMMs (ie, their relative duration) per assessment (median [IQR], 1.55% [0.64%-3.57%]).

### Initial Accuracy of the Algorithm

**Figure 2.**
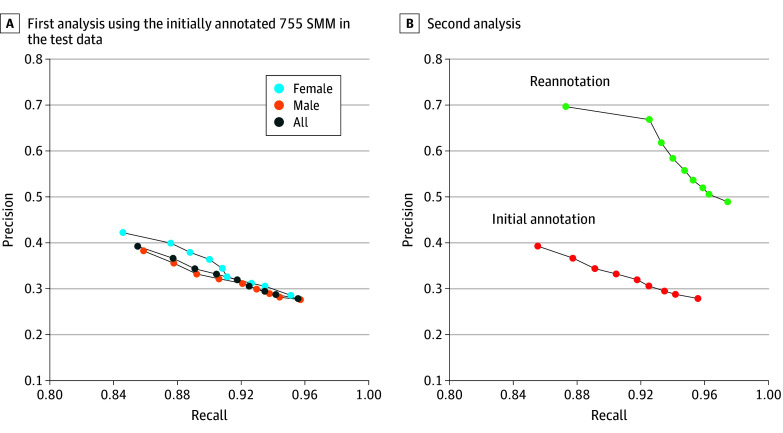
Algorithm Accuracy Precision and recall values demonstrate the accuracy of the algorithm in identifying SMMs on individual video frames in the test data composed of 24 assessments from 21 children. Each point represents precision and recall values when selecting a specific SMM confidence threshold between 0.5 and 0.9. A, This analysis was performed once with all children and again with male or female children only. B, Second analysis using the SMM segments that were reannotated by the 2 independent annotators who exhibited high test-retest reliability. Precision and recall values are presented for these video segments when using the initial manual labels (red) and again when using the correct reannotated labels (green). SMM indicates stereotypical motor movements.

After training the algorithm with data from 295 assessments of 220 children (see Methods), we tested its accuracy with independent data from 24 assessments of 21 children (Figure 2A). We performed this analysis once with all children and again while separating male (15 participants) and female (6 participants) children. The algorithm yielded a score between 0 and 1, representing its confidence about the presence of an SMM. We compared the algorithm’s accuracy per frame at confidence thresholds between 0.5 and 0.9. Using a threshold of 0.85 yielded precision and recall of 36.64% (95% CI, 29.97%-49.99%) and 87.72% (95% CI, 84.22%-94.22%) for the entire group, 35.56% (95% CI, 27.38%-52.60%) and 87.77% (95% CI, 81.73%-94.95%) for male children, and 39.93% (95% CI, 16.90%-62.99%) and 87.59% (95% CI, 82.33%-99.99%) for female children, respectively. The high recall values suggested that the algorithm accurately identified most manually annotated SMMs, but the low precision values indicated a high number of false positives. There were no significant differences in accuracy across male and female children at the 0.85 threshold.

### Reannotation and Interrater Reliability of the Test Data

The low precision of the algorithm suggested that there may have been video segments with SMMs that were missed by the initial manual annotation. Concurrently, we also wanted to assess interrater reliability, which was not examined in the initial annotation process. To achieve both, we performed a second round of manual annotation with 2 independent annotators (H.K. and O.H.). We extracted 1456 short video segments from the 24 assessments in the test set that contained an equal number of true positives (hits), false positives (false alarms), true negatives (correct rejections), and false negatives (misses) in terms of the match between algorithm labeling and the initial manual annotation. The 2 annotators, blind to the initial annotation and the algorithm’s labeling, manually reannotated these segments independently (ie, labeled each segment as containing an SMM or not). There was 90% agreement between the 2 annotators, corresponding to excellent interrater reliability (*κ* = 0.76).

Video segments identified as containing an SMM by either annotator were relabeled as SMMs. The reannotation process revealed that 51% of the video segments initially designated as false positives were actually true positives (ie, contained an SMM that was missed in the initial annotation), and 9.8% of the segments initially designated as false negatives were actually true negatives (ie, did not contain an SMM). We believe that the high percentage of missed SMMs demonstrates the difficulty of manually annotating SMMs within long video recordings of behavioral assessments. Testing the algorithm’s accuracy with the reannotated 1456 video segments revealed a final precision (or positive predictive value) of 66.82% (95% CI, 55.28%-72.05%) and recall (or sensitivity) of 92.53% (95% CI, 81.09%-95.10%), respectively, when using a threshold of 0.85 (Figure 2B). At this threshold, the algorithm achieved 95.45% (95% CI, 94.31%-96.91%) specificity and 99% (95% CI, 99%-100%) negative predictive value.

### Accuracy of SMM Quantification per Assessment and Child

**Figure 3.**
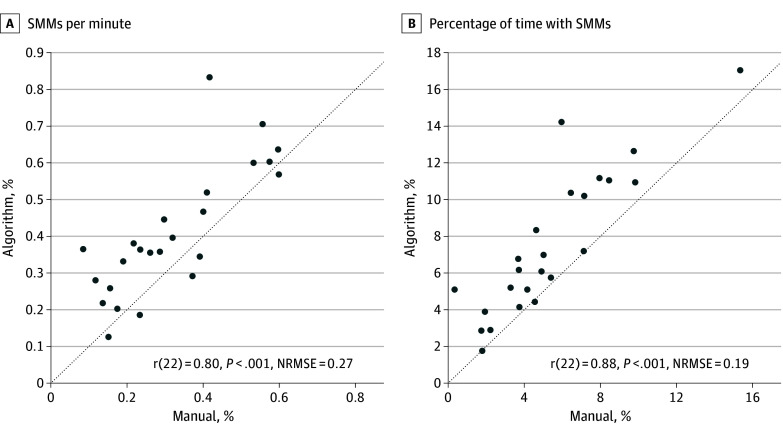
Accuracy in Quantifying Stereotypical Motor Movement (SMM) Severity Accuracy of the algorithm in quantifying SMMs per assessment. Scatterplots compare the number of SMMs per minute and percentage of time with SMMs as quantified by manual annotation vs the algorithm. Pearson correlation coefficients and their statistical significance are noted in each panel. The dotted line indicates serves as a reference line indicating perfect agreement between the manual annotator and the automated model.

While frame-wise precision recall curves are important for determining the accuracy of our algorithm in computer science terms, this assessment is overly conservative for basic and clinical autism research purposes where one is interested in quantifying the overall amount or rate of SMMs that a child exhibits. A more relevant accuracy test for such purposes is to compare the number and duration of SMMs exhibited by each child as quantified by the SMM algorithm vs manual annotation (Figure 3). The algorithm-derived measures were strongly and significantly correlated with manually annotated measures for both the number of SMMs per assessment (*r*_22_ = 0.80; 95% CI, 0.67-0.93; ρ_22_ = 0.80; concordance correlation coefficient [CCC] = 0.70; *P* < .001) and their percentage of time per assessment (*r*_22_ = 0.88; 95% CI, 0.74-0.96; ρ_22_ = 0.87; CCC = 0.73; *P* < .001) when using Pearson, Spearman, or CCC, respectively. Note that this analysis used the reannotated data described previously.

## Discussion

Our algorithm was able to identify or recall more than 90% of the video frames that were manually labeled as containing SMMs. Initially, our algorithm appeared to yield a high number of false positives (ie, low precision, Figure 3A), but comprehensive reannotation of 1456 video segments by 2 independent annotators, who exhibited 90% agreement and high interrater reliability, revealed that 51% of segments initially considered false positives were in fact true positive, thereby increasing precision from 37% to 67%. This demonstrated that the annotators missed many SMMs during their initial examination of the long, approximately 40-minute, assessment videos. These manual labeling errors were corrected only during reinspection of the video segments marked by our algorithm. This highlights the advantage of using an automated algorithm for detecting rare events within long video recordings that are difficult and boring to manually annotate. Note that the reannotation process was performed in a fair manner by including an equal number of true and false positive and true and false negative segments in the 1456 reexamined segments.

Most importantly, strong, significant correlations were evident between algorithm-derived and manually annotated SMM rates and durations per assessment (ie, the number of SMMs per minute and the proportion of time with SMMs) (Figure 3). These correlations demonstrate the value of our algorithm for quantifying SMM severity per child. We believe our algorithm has the potential to replace manual annotation techniques previously applied to short recordings in small samples,^6,8,20,21^ thereby enabling large scale studies on a variety of topics, such as characterizing the development of SMMs in children with ASD and identifying their behavioral, physiological,^12^ and neural triggers.

### Previous SMM-Related Algorithms and the Current Algorithm

Our algorithm is novel and distinct from previous computer vision algorithms developed for SMM classification. Our algorithm can scan long video recordings of behavioral assessments and detect segments that contain a wide variety of heterogeneous SMMs (see eTable 1 in Supplement 1). In contrast, previously published algorithms were trained with short, approximately 90-second, home-videos^27,28,29^ or Kinect recordings^26,28^ to distinguish between 3 to 5 specific types of predefined SMMs and cannot identify heterogeneous, sparse SMMs within extensive real-life videos.

### Previous SMM-Related Datasets and ASDPose

Previous examples of SMM datasets include curated short home videos of SMMs that were recorded by parents of children with ASD and posted online. The original Self-Stimulatory Behavior Dataset^43^ includes 75 videos (approximately 90 seconds long) containing examples of arm flapping, head banging, or spinning SMMs. The Expanded Stereotype Behavior Dataset^29^ includes 141 videos (approximately 20 seconds long) containing spinning, arm flapping, hand action, and head banging SMMs. Neither dataset contains any clinical or demographic information about the recorded children, who may or may not have a formal diagnosis of ASD, and most recordings contain only the exhibited SMM.

In contrast, the ASDPose dataset contains the skeletal representation of children with ASD in extensive (approximately 40 minutes long) recordings from 319 behavioral assessments of 241 children with thorough clinical characterization. The dataset includes demographic information as well as ADOS-2 scores for all the children and cognitive and PLS-4 scores for most. However, to maintain privacy, ASDPose does not include the raw video. The dataset is released along with the manual annotation of 7352 SMM segments and details about our selection of training and testing data for transparency and reproducibility.

### Limitations

Our study had limitations. General limitations to assessing SMMs in video recordings include the necessity for substantial computational power, storage capacity, and overcoming privacy limitations. In addition, the current version of our algorithm has several specific limitations. First, it was trained only with recordings from behavioral assessments of children with ASD aged 1.4 to 8.0 years, and may therefore produce less accurate results with recordings from other clinical or experimental contexts, older children, typically developing children, or children with other developmental conditions. Moreover, extending the number of recordings from female children with ASD would be beneficial for identifying female-specific SMMs. Second, the current algorithm was not trained to classify between different types of SMMs; instead it was trained to identify any SMM, regardless of its type. While this may be considered a limitation, we believe it is also a feature that enables users to identify a wide range of SMMs without committing to a specific SMM categorization system, of which there are several. Third, we did not assess the accuracy of algorithm-defined SMM onset and offset times relative to manual labeling. Moreover, developing additional measures of SMM intensity and severity would also be highly warranted. All these limitations will be addressed in future versions of our algorithm as we and others train and test it on additional data.

## Conclusions

Our algorithm and ASDPose dataset offer an innovative way of studying SMMs in ASD and other disorders where individuals exhibit SMMs. This novel digital phenotyping technique offers opportunities for studying the natural history of SMMs in autism as well as their underlying neural and physiological mechanisms. Future versions of our algorithm will extend its utility, robustness, and applicability even further.

## Notes

Supplement 1. **eFigure 1. **Manual Annotation **eFigure 2. **Using YOLOv5 to Identify Child and Adult Skeletons **eFigure 3. **Schematic of Algorithm Pipeline and Scoring **eFigure 4. **Distribution of Manually Annotated SMM Categories **eTable 1.** Description of the SMM Categories That Were Manually Annotated in the Current Study **eTable 2. **Descriptive Statistics of Manually Annotated Video Recordings **eTable 3. **Comparison of the Test Set Accuracy Scores, Mean Class Accuracy Scores, Precision, Recall, and Epoch Time
eReferences

Supplement 2.
Data Sharing Statement
