# Supplementary material for: Automated Analysis of Stereotypical Movements in Videos of Children With Autism Spectrum Disorder
Source: JAMA Netw Open. 2024 Sep 12;7(9):e2432851. doi: 10.1001/jamanetworkopen.2024.32851 (PMC11393723; doi:10.1001/jamanetworkopen.2024.32851)
Supplement: Supplement 1. — eFigure 1. Manual Annotation eFigure 2. Using YOLOv5 to Identify Child and Adult Skeletons eFigure 3. Schematic of Algorithm Pipeline and Scoring eFigure 4. Distribution of Manually Annotated SMM Categories eTable 1. Description of the SMM Categories That Were Manually Annotated in the Current Study eTable 2. Descriptive Statistics of Manually Annotated Video Recordings eTable 3. Comparison of the Test Set Accuracy Scores, Mean Class Accuracy Scores, Precision, Recall, and Epoch Time eReferences [file jamanetwopen-e2432851-s001.pdf]

Supplemental Online Content

Barami T, Manelis-Baram L, Kaiser H, et al. Automated analysis of stereotypical movements in videos of children with autism spectrum disorder. *JAMA Netw Open*. 2024;7(9):e2432851. doi:10.1001/jamanetworkopen.2024.32851

eFigure 1. Manual Annotation

eFigure 2. Using YOLOv5 to Identify Child and Adult Skeletons

eFigure 3. Schematic of Algorithm Pipeline and Scoring

eFigure 4. Distribution of Manually Annotated SMM Categories

eTable 1. Description of the SMM Categories That Were Manually Annotated in the Current Study

eTable 2. Descriptive Statistics of Manually Annotated Video Recordings

eTable 3. Comparison of the Test Set Accuracy Scores, Mean Class Accuracy Scores, Precision, Recall, and Epoch Time

eReferences

This supplemental material has been provided by the authors to give readers additional information about their work.

© 2024 Barami T et al. *JAMA Network Open.*

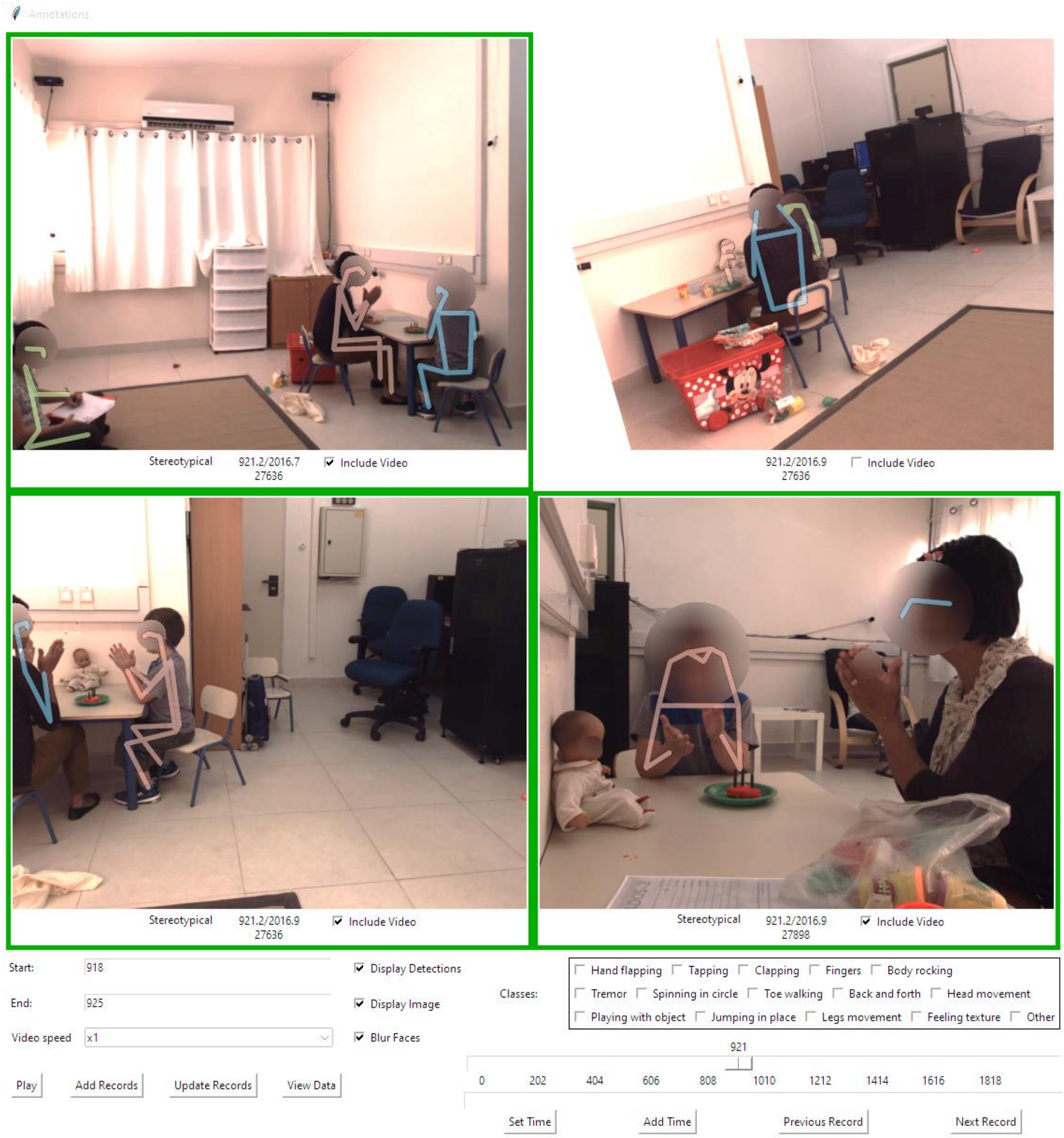

eFigure 1. Manual Annotation was performed with in-house developed software that enabled annotators to quickly mark the start and end times of SMMs, the child's skeleton number, and the cameras (i.e., views) where the child was visible.

© 2024 Barami T et al. *JAMA Network Open.*

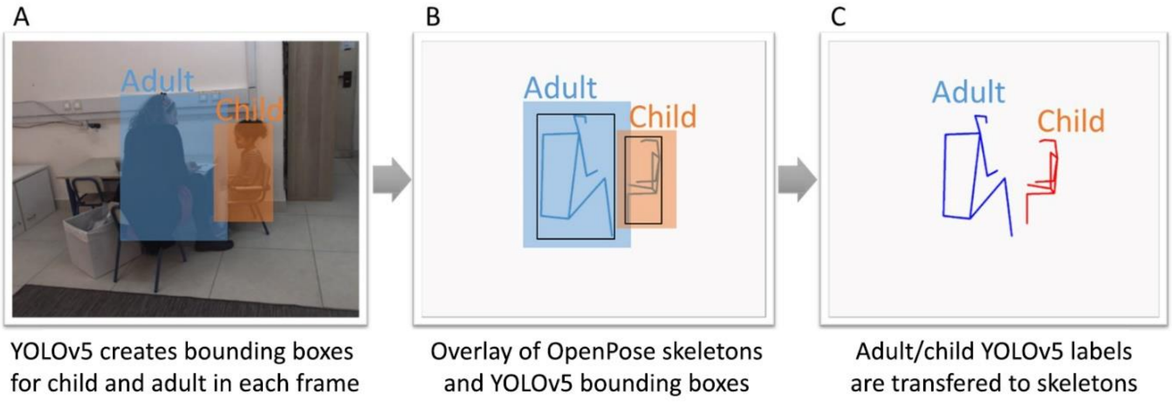

eFigure 2. Using YOLOv5 to Identify Child and Adult Skeletons. (A) The YOLOv5 object detection model was trained to identify all children and adults in each video frame. The algorithm marks the location of each with a rectangular bounding box. (B) Overlay of the YOLOv5 bounding boxes and the extracted OpenPose skeletons for the same video frame. Black rectangles are bounding boxes of each skeleton. (C) YOLOv5 child/adult labels are transferred to the OpenPose skeleton. The identity of each skeleton is determined by maximizing the intersection-over-union of the YOLOv5 and OpenPose bounding boxes. YOLOv5 bounding boxes that do not match any OpenPose skeleton are discarded, and skeletons with no matching YOLOv5 bounding box are labeled as Adult.

© 2024 Barami T et al. *JAMA Network Open.*

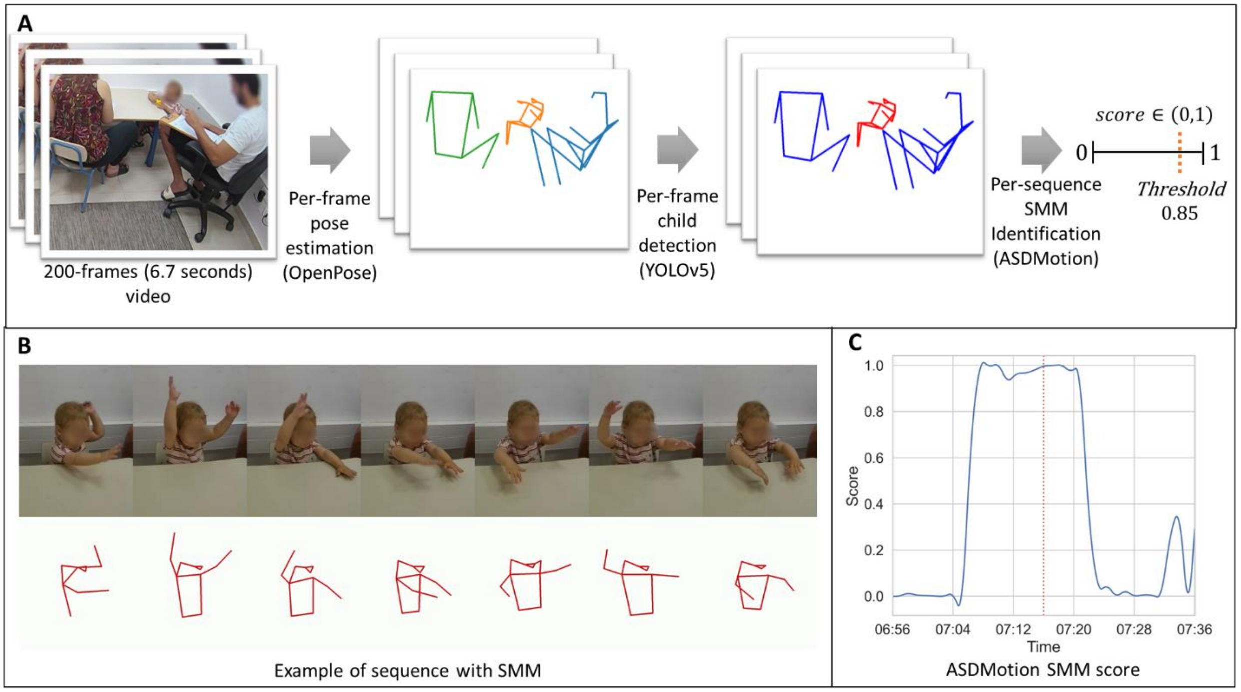

eFigure 3. Schematic of Algorithm Pipeline and Scoring. (A) Example of a 200-frame video segment that is processed with the pose estimation algorithm (OpenPose), then with the child detection algorithm (YOLOv5), and finally the child's skeletal representation is analyzed by ASDMotion, yielding an SMM score between 0 and 1. (B) Example video and corresponding skeleton sequence where a child exhibits an SMM. (C) ASDMotion's output for a 40-second video containing the SMM sequence in B. SMM scores are computed per 200-frame sliding window (6.7 seconds) with a 30 frame (1 second) step size, yielding a time-course of scores per video.

© 2024 Barami T et al. *JAMA Network Open.*

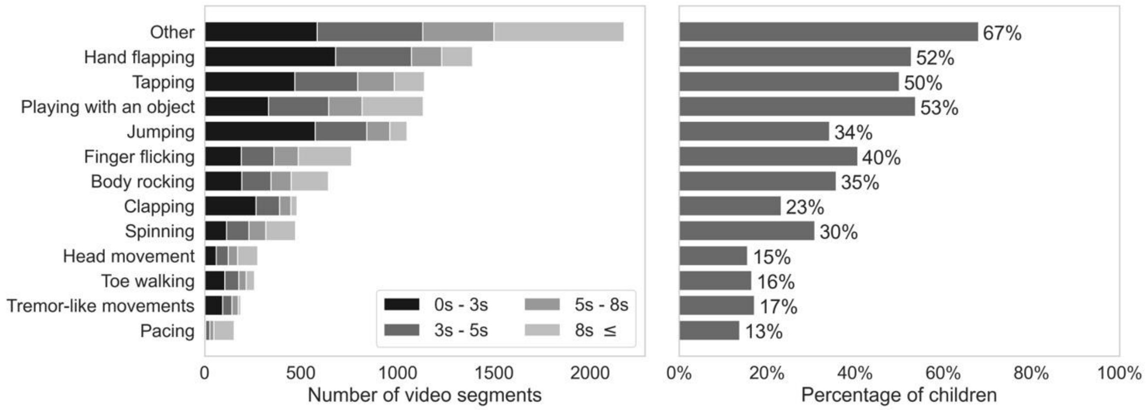

eFigure 4. Distribution of Manually Annotated SMM Categories. (A) Number and lengths of segments manually annotated as belonging to each category. (B) Percentage of children who exhibited an SMM in each category at least once. Note that most children exhibited more than one type of SMM. When training and testing ASDMotion, we combined all movement types, lumping all SMMs across all categories together.

© 2024 Barami T et al. *JAMA Network Open.*

eTable 1. Description of the SMM Categories That Were Manually Annotated in the Current Study. Categories were defined according to the physical characteristics of the movements. When training and testing ASDMotion, we combined all movement types, lumping all SMMs across all categories together.

Category	**Description**
Clapping	Striking palms of hands against one another
Hand flapping	Flapping hands by moving the wrists or elbows up and down or side to side in the air
Finger flicking	Moving fingers in the air in a repetitive manner
Tapping	Repeatedly tapping a surface with the hand or fingers
Spinning	Spinning in circles while standing
Pacing	Walking back and forth along the same path
Jumping	Jumping or hopping repeatedly in the same spot or around the room
Toe walking	Walking on the toes instead of flat feet
Body rocking	Swaying the body back and forth or side to side while sitting or standing
Tremor-like movements	Rhythmic muscle contractions and relaxations yielding repetitive twitching movements of one or more body parts
Playing with an object	Using a toy in an unusual and repetitive manner. For example, repeatedly licking a ball or repeatedly spinning the wheel of a toy car.
Head movement	Rhythmic head movement from side-to-side or up-and-down
Other	Unique movements that were observed rarely and did not fit one of the categories above. For example, repeatedly rotating a yamaka, playing with hair in a peculiar way, repeatedly touching a point on the wall, etc..

eTable 2. Descriptive Statistics of Manually Annotated Video Recordings.

	Median	***Inter Quartile Range***
Assessment videos length (minutes)	35.9	30.6-48.21
Average duration of an SMM (minutes)	0.13	0.08-0.19
Percentage of video time with SMMs	1.4%	0.63%-3.21%
Total number of SMMs per video	4	2-7
SMM frequency per minute	0.1	0.05-0.2

The PoseConv3D model and comparison with alternative models for SMM identification

ASDMotion is based on a PoseConv3D model that was pretrained (i.e., fine-tunned) with the Kinetics-400 dataset[4]. The PoseConv3D model uses a 3D heatmap volume of the skeletal movements as input to a 3D convolutional neural network (CNN), which was trained to identify SMMs. Each 3D heatmap is composed of 48 2D heatmaps (one per video frame) with the x,y locations of the 17 skeleton joints, represented with the OpenPose estimation confidence value for that given joint (i.e., “heat” value per joint). Hence the 3D heatmap represents both the skeleton movement and the OpenPose confidence in estimating the skeleton position for the given 48-fame segment. This architecture was specifically designed to identify spatial and temporal dependencies in human skeleton movements over time (i.e., compact representations of human body movements). The algorithm was trained with samples from the training dataset that were 200-frames long (i.e., 6.7 seconds). Training samples had a batch size of 64 input skeleton sequences (containing sequences with and without SMMs) and the training process utilized a stochastic gradient descent optimization for 100 epochs (83.49 minutes per epoch).

We examined three alternative models before selecting the PoseC3D Kinetics-400 model for the analyses presented in the main text. The three alternative models were: PoseC3D

© 2024 Barami T et al. *JAMA Network Open.*

(without fine-tunning), Spatio-Temporal Graph Convolutional Network (ST-GCN), and Two-stream Adaptive Graph Convolutional Network (2S-AGCN).

ST-GCN[1] is a graph convolutional network which takes a 2D skeleton input and constructs a spatial-temporal graph. This graph represents the human movement with the nodes representing the joints, one set of edges representing the spatial relationship across nodes (i.e., skeleton shape) and another set of edges representing the change/movement of joints/nodes across frames (i.e., time). Two-stream Adaptive Graph Convolutional Network (2S-AGCN)[2] is another graph-convolutional-based network that models joint position movements and skeleton segment (i.e., "bone") movements separately by two independent networks whose output is summed to generate a final prediction.

Comparison of the four models was performed using the same train and test datasets described in the main text. We first trained each of the models with data from 295 assessments of 220 ASD children and then tested their performance with an independent test set from video recordings of 24 assessments of 21 children. We also compared the run time for training per epoch to measure the computational cost of each algorithm.

While the PoseC3D fine-tuned with Kinetics-400 model was the most time-consuming, it also provided the highest accuracy, which motivated us to select it for the current study.

eTable 3. Comparison of the Test Set Accuracy Scores, Mean Class Accuracy Scores, Precision, Recall, and Epoch Time when comparing the PoseC3D, PoseC3D with Kinetics-400 pretraining, ST-GCN, and 2S-AGCN models.

Model	**Precision**	**Recall**	**Epoch time (minutes)**
PoseC3D	55.73%	85.31%	77.10
PoseC3D K-400	66.82%	92.53%	83.49
ST-GCN	25.15%	77.91%	35.16
2S-AGCN	45.75%	74.71%	39.88

© 2024 Barami T et al. *JAMA Network Open.*

eReferences

1. Yan S, Xiong Y, Lin D. Spatial Temporal Graph Convolutional Networks for Skeleton-Based Action Recognition. Published online January 23, 2018. http://arxiv.org/abs/1801.07455

2. Shi L, Zhang Y, Cheng J, Lu H. Two-Stream Adaptive Graph Convolutional Networks for Skeleton-Based Action Recognition. Published online May 19, 2018. http://arxiv.org/abs/1805.07694

4. Kay W, Carreira J, Simonyan K, et al. The Kinetics Human Action Video Dataset. Published online May 19, 2017. http://arxiv.org/abs/1705.06950

© 2024 Barami T et al. *JAMA Network Open.*
