# Supplementary material for: Automated Analysis of Stereotypical Movements in Videos of Children With Autism Spectrum Disorder
Source: JAMA Netw Open. 2024 Sep 12;7(9):e2432851. doi: 10.1001/jamanetworkopen.2024.32851 (PMC11393723; doi:10.1001/jamanetworkopen.2024.32851)
Supplement: Supplement 2. — Data Sharing Statement [file jamanetwopen-e2432851-s002.pdf]

Data Sharing Statement

Barami. Automated Analysis of Stereotypical Movements in Videos of Children With Autism Spectrum Disorder. *JAMA Netw Open*. Published September 12, 2024.
doi:10.1001/jamanetworkopen.2024.32851

Data
Data available: Yes
Data types: Deidentified participant data
How to access data: Source for training and executing the algorithm:
https://github.com/Dinstein-Lab/ASDMotion
When available: With publication

Supporting Documents
Document types: Statistical/analytic code
How to access documents: https://github.com/Dinstein-Lab/ASDMotion
When available: With publication

Additional Information
Who can access the data: For everyone.
Types of analyses: For any purpose.
Mechanisms of data availability: Online. https://github.com/Dinstein-Lab/ASDMotion
